# A homozygous missense variant in *DND1* causes non-obstructive azoospermia in humans

**DOI:** 10.3389/fgene.2022.1017302

**Published:** 2022-09-30

**Authors:** Xuefeng Xie, Mazhar Khan, Muhammad Zubair, Abbas Khan, Ranjha Khan, Jianteng Zhou, Yuanwei Zhang, Muzafar Said, Sher Ali Khan, Qamar Zaman, Ghulam Murtaza, Muzamil Ahmad Khan, Wei Liu, Xiaoning Hou, Huan Zhang, Bo Xu, Xiaohua Jiang, Shun Bai, Qinghua Shi

**Affiliations:** ^1^ The First Affiliated Hospital of USTC, Hefei National Laboratory for Physical Sciences at Microscale, The CAS Key Laboratory of Innate Immunity and Chronic Disease, School of Basic Medical Sciences, Division of Life Sciences and Medicine, CAS Center for Excellence in Molecular Cell Science, Collaborative Innovation Center of Genetics and Development, University of Science and Technology of China, Hefei, China; ^2^ Department of Bioinformatics and Biological Statistics, School of Life Sciences and Biotechnology, Shanghai Jiao Tong University, Shanghai, China; ^3^ Malka Andrology, Fertility and IVF Center, Roshan Specialized Hospital, saidu sharif, Pakistan; ^4^ Gomal Centre of Biochemistry and Biotechnology, Gomal University, Dera Ismail Khan, Pakistan

**Keywords:** DND1, male infertility, NOA, gene mutation, homozygous missense mutation

## Abstract

Non-obstructive azoospermia (NOA) is a severe factor of male infertility; it affects approximately 1% of the global male population and accounts for 40% of male infertility cases. However, the majority of NOA cases remain idiopathic. This is the first study using whole-exome sequencing (WES) to identify a novel missense mutation in the *DND1* gene (c.212A>C, p. E71A) from a Pakistani family, that includes three males with NOA. This mutation is predicted to cause DND1 protein misfolding and weaken the DND1 interaction with NANOS2, a significant regulator in primordial germ cell development. Our study identified a *DND1* pathogenic mutation in NOA patients and highlighted its critical role in male fertility in humans.

## Introduction

Approximately 15% of couples worldwide experience infertility, while male infertility-associated factors are prevalent in half of these couples (Inhorn and Patrizio, 2015; Krausz and Riera-Escamilla, 2018). Azoospermia, the absence of sperm in the ejaculate, affects 10–20% of men with infertility and is classified as either obstructive azoospermia (OA) or non-obstructive azoospermia (NOA) (Peña et al., 2020). NOA is characterized by the absence or reduction of germ cells and is mainly caused by genetic defects, testicular tumors, and chemotherapy (Kasak and Laan, 2021). Mutations in testis-enriched genes, including mitosis genes (*DAZL, NANOS1*) and meiosis genes (*DMC1*, *STAG3*, *C14ORF39*, and *MEIOB*) have been implicated in NOA (Teng et al., 2002; Kusz-Zamelczyk et al., 2013; He et al., 2018; Gershoni et al., 2019; van der Bijl et al., 2019; Fan et al., 2021). However, genetic factors underlying the majority of NOA cases require further investigation.

The dead end 1 (*DND1*) gene encodes a protein that binds RNA and plays an essential role in gene regulation in biological activities, including embryonic development, cell differentiation, cell cycle, cell growth and apoptosis, and its dysfunction can result in a variety of diseases (Das et al., 2020). In mice, DND1 plays a crucial role in maintaining the population of primordial germ cells and suppressing tumors derived from germ cells (Noguchi and Noguchi, 1985; Sakurai et al., 1995; Youngren et al., 2005; Cook et al., 2009; Northrup et al., 2012). Previous studies have shown that DND1 is essential for the binding of target RNAs by NANOS2, a major regulatory factor in spermatogenesis (Suzuki et al., 2016; Yamaji et al., 2017; Zealy et al., 2017). Mutation in *Dnd1* disrupted the interaction between DND1 and its partners, resulting in abnormal differentiation of male germ cells and male infertility in mice (Li et al., 2018). However, the function of the DND1 protein in human male fertility remains unknown.

Here, for the first time, we identified a homozygous missense mutation in *DND1* (c.212A>C, p. E71A) from three males suffering from NOA in a Pakistani family. *In vitro* experiments showed that the mutation caused a remarkable decrease in DND1 protein levels and affected its interaction with NANOS2. These data provide functional evidence that pathogenic variants in *DND1* cause male infertility in humans.

## Materials and methods

### Patients

**FIGURE 1:**
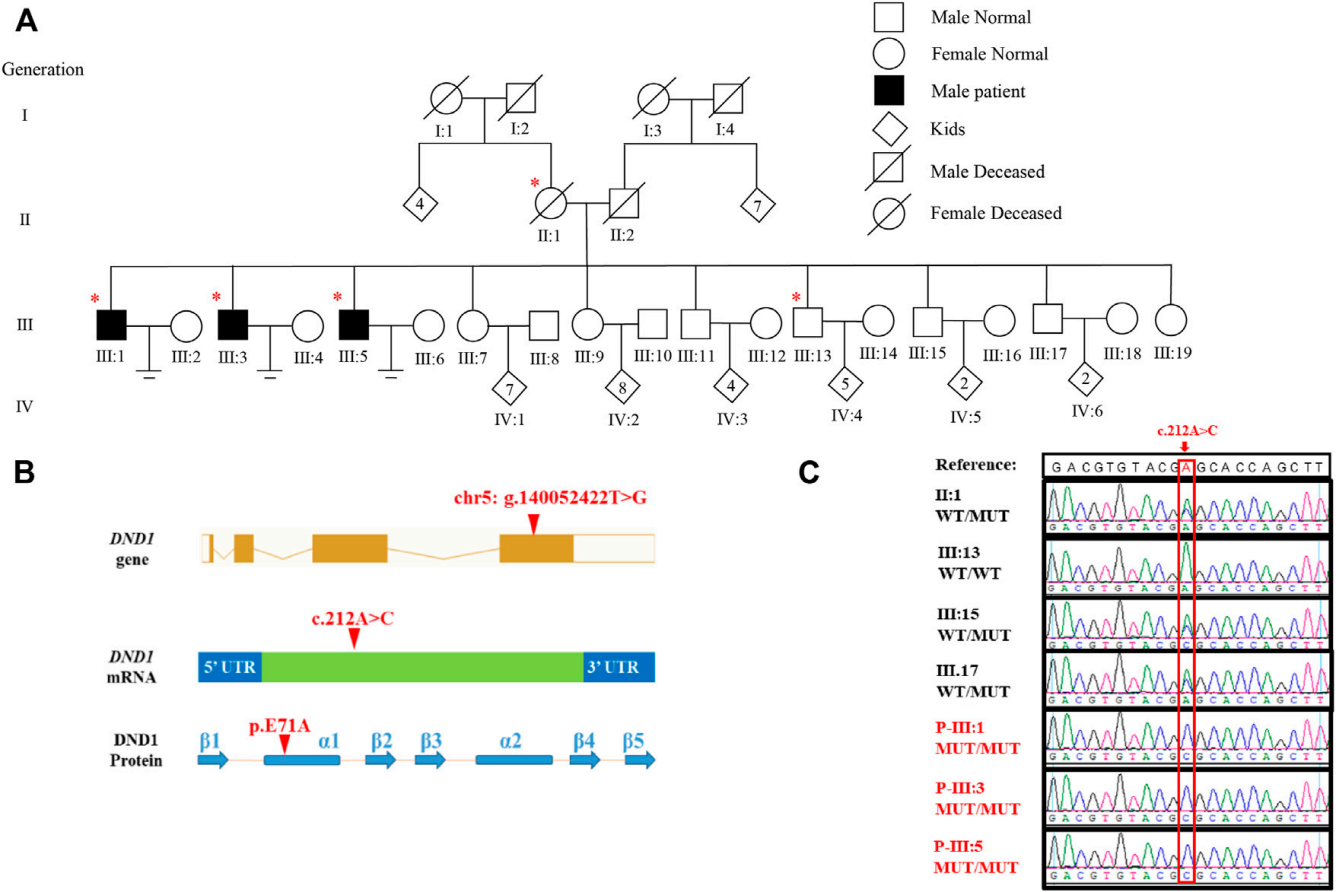
Identification of DND1 E71A Mutation in three patients with azoospermia. **(A)** Pedigree of the affected family. Circles and squares denote females and males, respectively. Deceased family members are denoted by slash symbols. Individuals marked with asterisks, undergone whole exome sequencing. **(B)** Location of the identified variant in *DND1* at both cDNA and protein levels. The mutation site was located in α-subunit of RBD1. **(C)** Chromatograms of confirming *DND1* mutation site by Sanger sequencing. WT, wild-type allele. MT, mutant allele. A, adenine; C, cytosine; G, guanine; T, thymine.

We recruited three males with primary infertility from a large family (Figure 1A). Each participant completed a detailed questionnaire regarding his reproductive history. According to clinical investigations, all of the affected individuals displayed normal erection and ejaculation, male external genitalia and secondary sexual characteristics. Semen parameters including sperm concentration, motility, and morphology were measured according to the World Health Organization (WHO) criteria for human semen analysis (Boitrelle et al., 2021). Blood samples were collected from all the available family members for genomic DNA extraction. This study was approved by the Institutional Ethical Committee of the University of Science and Technology of China (USTCEC202000003). Each family member provided written consent prior to the collection of biological samples.

### Gene filtration through whole-exome sequencing and sanger sequencing

Total gDNA from peripheral blood of the patients and their family members was obtained using the QIAamp DNA Blood Mini Kit (QIAGEN) according to the manufacturer’s instructions. Exons were enriched *via* the Agilent SureSelect Human All Exon V5 Kit. The enriched DNA was sequenced on the Illumina HiSeq 2000 platform (Illumina, San Diego, CA). Sequencing reads (.qseq format) were aligned to the reference human genome (hg19) using Burrows-Wheeler Aligner (BWA) software with default parameters. SAMtool (http://samtools.sourceforge.net/) was used to convert the SAM files from each sample into BAM files. The PCR duplicates were removed with the Picard tool (http://picard.sourceforge.net/). Files were further processed with the Genome Analysis Toolkit (GATK) available from the Broad Institute (http://www.broadinstitute.org/gatk/), followed by realignment through indel realigner and variants (single-nucleotide variants (SNVs) and indels) calling using GATK’s Unified Genotyper (Zubair et al., 2022). PCR amplification and Sanger sequencing were performed to confirm the candidate pathogenic variants from whole-exome sequencing (WES). All primer sequences used for PCR and sequencing are shown in Supplementary Table S3.

### Phylogenetic analysis and structural modelling of DND1

The evolutionary conservation of altered amino acid residues was performed with MEGA (Tamura et al., 2013). The effect of pathogenic missense variants on protein structure was predicted by the SWISS-MODEL online tool.

### Plasmid construction and cell culture

The coding sequences of wild-type (WT) and mutant (MT) *DND1* were fused to EGFP. Human embryonic kidney 293T cells (HEK293T) obtained from ATCC (CRL-1573) were cultured in DMEM (VivaCell, C3113-0500) supplemented with 10% fetal bovine serum (FBS, Gibco, 16,000-044) and penicillin-streptomycin (Gibco, 15,140-122). Plasmids harboring WT or MT eGFP-DND1 were transfected into HEK293T cells using Lipofectamine 3000 according to the manufacturer’s instructions (Invitrogen, L3000015). At 36 h post-transfection, plasmid expression was examined under a Nikon ECLIPSE 80i microscope. At 44 h post-transfection, the cells were harvested for Western blotting analysis.

### Western blotting

The HEK293T cells were lysed in 4X Bolt LDS Sample Buffer (Invitrogen, B0008) with NuPAGE Antioxidant (Invitrogen, NP0005). The lysates were denatured for 10 min and then were separated by sodium dodecyl sulfate-polyacrylamide gel electrophoresis, followed by transferring the proteins to 0.45 μm pore size immobilon-P membranes (Millipore, IPVH00010). Membranes were blocked in TBST buffer (50 mM Tris, pH 7.4, 150 mm NaCl and 0.1% Tween-20) containing 5% nonfat milk for 1 h and incubated with primary antibodies (GFP, Abmart, M20004L; Myc, Abmart, M20002; mCherry, Abcam, AB125096; mCherry, Abmart, AB167453; β-Actin, Abcam, ab8227) at 4°C overnight. Following incubation with horseradish peroxidase (HRP)-conjugated secondary antibodies (HRP Donkey anti-rabbit IgG, BioLegend, Inc., 406,401; HRP Goat anti-mouse IgG, BioLegend, Inc., 405,306) for 1 h, the membranes were developed with chemiluminescence substrate by ImageQuant LAS 4000 imaging system (GE Healthcare).

### Co-immunoprecipitation (Co-IP)

The HEK293T cells were lysed in lysis buffer (50 mm Tris, pH 7.5, 150 mm NaCl, 5 mm EDTA and 0.1% NP-40) supplemented with PMSF, Protease Inhibitor Cocktail and MG132, followed by rotation at 4°C for 20 min. After centrifugation, 50 μL of the supernatant was taken out as the input. Beads (Pierce™ anti c-Myc beads, Thermo scientific, 88,842; Protein A/G Magnetic beads, bimake, B23202) were washed with pre-cooled lysis buffer for three times, then the beads were incubated with supernatant at 4°C for 2–3 h. After washing with lysis buffer for 6 times, the beads were boiled in 2×SDS sample buffer for 10 min. The samples were either analyzed by Western blotting immediately as described above or stored at −80°C.

## Results

### Clinical characteristics of three patients with azoospermia

In this study, we investigated a Pakistani family, having three infertile brothers suffering from NOA and male infertility (Figure 1A). The parents (II:1 and II:2) had three daughters and seven sons. The unmarried sister (III:19, 26 years old) had normal menstrual cycles and the two married sisters had six and five children, respectively. Among the seven brothers, four had children, whereas III:1 (43 years old), III:3 (36 years old) and III:5 (34 years old) were infertile despite being married for 22, 17, and 18 years, respectively.

The patients had no history of drinking, smoking, exposure to toxic chemicals, and any other diseases. The clinical information of each patient is summarized in Table 1. Routine semen analyses revealed that no spermatozoa were detected in their ejaculates, although the semen volume was normal. Ultrasound examination revealed normal vas deferens and small testes in all patients (right: 3.5*1.7*2.8 cm^3^ and left: 3.6*2.0*2.6 cm^3^ for III:1; right: 3.8*2.3*2.6 cm^3^ and left: 4.0*2.3*2.6 cm^3^ for III:3; right: 3.8*2.3*2.6 cm^3^ and left: 4.0*2.4*2.7 cm^3^ for III:5). Additionally, all enrolled patients had normal sex hormone levels and a 46, XY karyotype. Together, azoospermia in those patients is caused by spermatogenic failure rather than obstruction (Esteves, 2015).

**TABLE 1:** Clinical Investigations of patients harboring novel homozygous *DND1* variant.

	References values	III:1	III:3	III:5
Age (years)	-	43	36	34
Gender	-	Male	Male	Male
Height/weight (cm/kg)	-	185/70	175/87	175/62
Semen volume (ml)	>1.4 ml	2.0 ml	2.5 ml	2.8 ml
Sperm count (millions/ml)	>16 million/ml	0	0	0
Testosterone (ng/dl)	262-870	690.1	N/A	350.1
FSH (mIU/ml)	2.1–18.6	11.3	N/A	8.9
LH (mIU/ml)	1.7–11.2	9.9	9.0	7.0
Prolactin (ng/ml)	3.6–16.6	7.5	9.5	14.7
Estradiol (pg/ml)	<75 pg/ml	2.0	8.5	9.13
Progesterone (pg/ml)	<0.46 ng/ml	0.11	0.31	0.44

a Reference values were published in WHO (2021).

### Identification of a missense variant in *DND1*

To determine the genetic cause of azoospermia in these patients, WES was conducted in the three affected individuals (III:1, III:3, and III:5), their mother (II:1) and the healthy brother (III:13) (Figure 1A) using gDNA extracted from peripheral blood lymphocytes. Analysis of WES data identified three candidate pathogenic variants: *PCDHGA8* (c. 862C>T, p. Q288X), *ARHGAP26* (c. 2137G>A, p. V713I) and *DND1* (c.212A>C, p. E71A), of which only *DND1* is a highly testis-expressed gene (Supplementary Table S1) and its homozygous deficiency cause male infertility (Seelow et al., 2009; Zhang et al., 2013; Li et al., 2018; Niimi et al., 2019). Details regarding the exclusion of the two genes can be found in Supplementary Table S2. The autosomal recessive inheritance pattern of the missense variant of *DND1* in this family was confirmed by Sanger sequencing (Figure 1C). In addition, the mutation was not present in several general population frequency databases, such as the 1000 Genomes Project and Genome Aggregation Database (gnomAD) (Supplementary Table S1).

### The mutated amino acid in *DND1* (c.212A>C, p.E71A) is highly conserved

**FIGURE 2:**
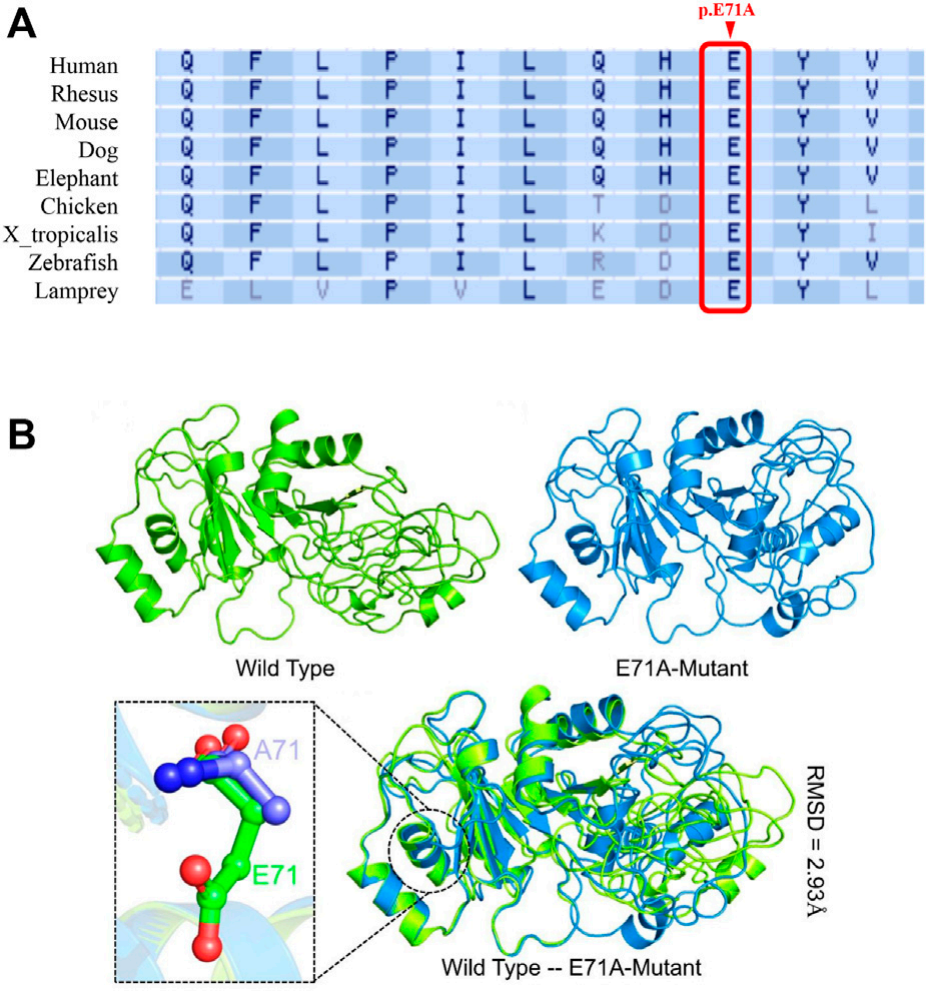
*DND1* E71 is highly conserved, and the E71A mutation is likely to be pathogenic. **(A)** Multiple protein sequence alignment shows that the affected amino acid across species was highly conserved. Arrowheads, the mutation site. **(B)** The predicted structure of the DND1 protein. The wild type structure is shown in green while the E71A mutant is given in marine blue. The mutated residue is also shown as stick in the lower panel. As predicted, the E71 is exposed to the surface of the α-subunit and E71A the mutant may reduce protein stability.

*DND1* gene is composed of four coding exons and produces 353 amino acids containing two consecutive RNA-binding domains (RBDs) and one double-strand RBD (dsRBD) with five β-subunits and two α-subunits. The variant p. E71A is located in the α-subunit of RBD1 (Figure 1B). Multiple sequence alignment of the DND1 protein showed the amino acid site of variant (p.E71A) is highly conserved across the eutherian species (Figure 2A). We further performed an *in silico* analysis to predict the structural changes in the DND1 protein (Figure 2B). As shown in Figure 2B (lower panel), disruption of the α-subunit was found in the DND1 mutated protein.

### DND1 E71A mutation altered DND1 localization and expression

**FIGURE 3:**
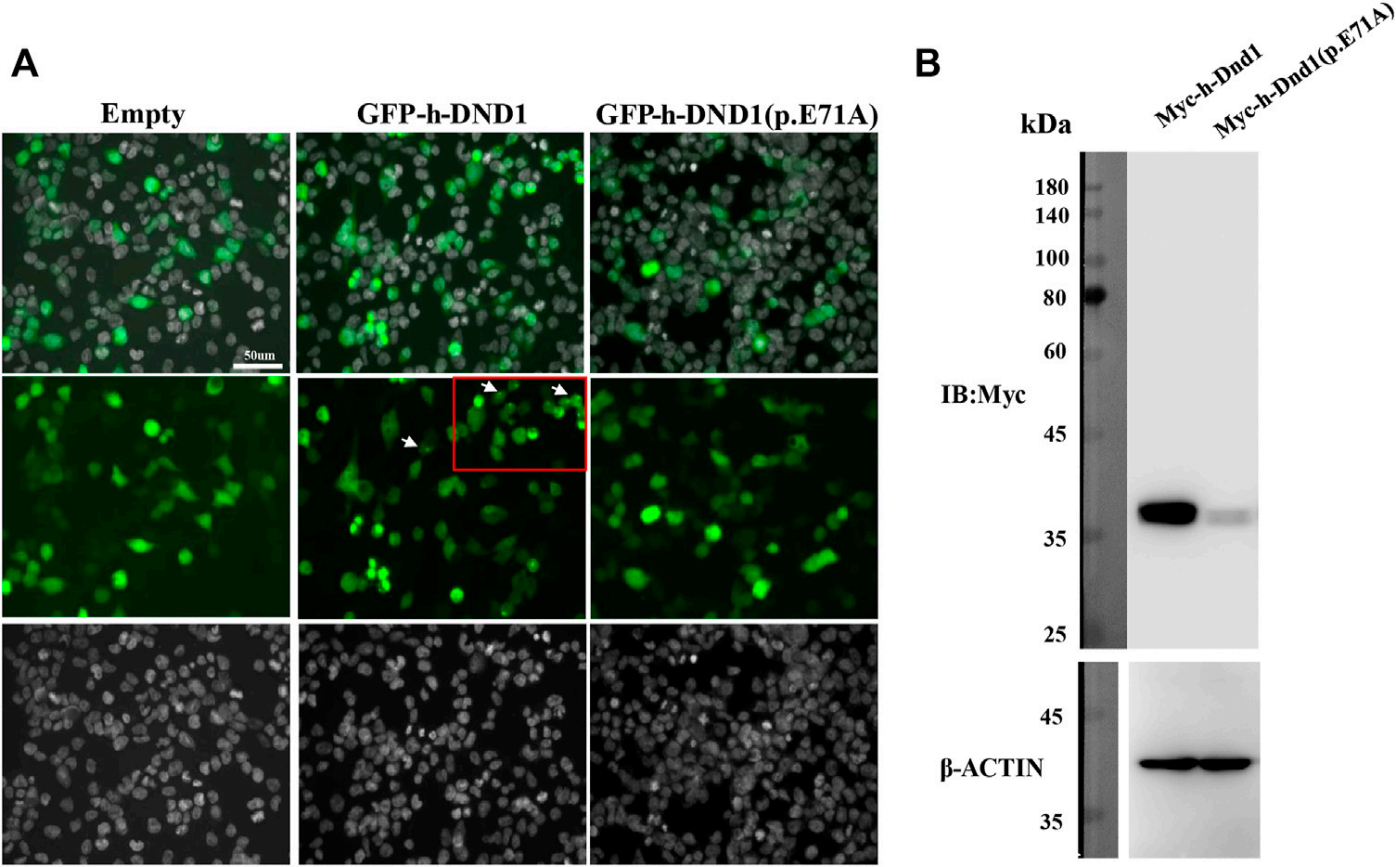
E71A mutation affected DND1 subcellular localization and protein expression. **(A)** Heterologous expression of human DND1 protein. GFP-fused wild-type and mutated DND1 (E71A) plasmids were transfected into in HEK293T cells. Green, GFP fluorescence. Cell nuclei were stained with DAPI (gray). Scale bar: 50 µm. **(B)** The expression levels of wild-type and mutant DND1 protein were detected by immunoblotting with an anti-GFP antibody. ACTIN served as a loading control.

To investigate the functional consequences of the *DND1* missense variant, the wildtype (WT) and mutant (MT) DND1 protein with N-terminal EGFP-tags were expressed transiently in HEK293T cells. We found that the E71A mutant exhibited reduced DND1 signal intensity in both the cytoplasm and the nucleus (Figure 3A) and our results are consistent with previous findings, where by using the single guide RNA (sgRNA) and enhanced third generation base editing system (4B2N1) in oocytes and by generating mutant mice targeting *Dnd1* gene with base mutation completely depleted PGCs and affected the protein-protein interaction of DND1 (Li et al., 2018). Moreover, the decrease in MT DND1 protein expression was confirmed by immunoblotting (Figure 3B).

### DND1 E71A mutation disrupted its interactions with NANOS2

**FIGURE 4:**
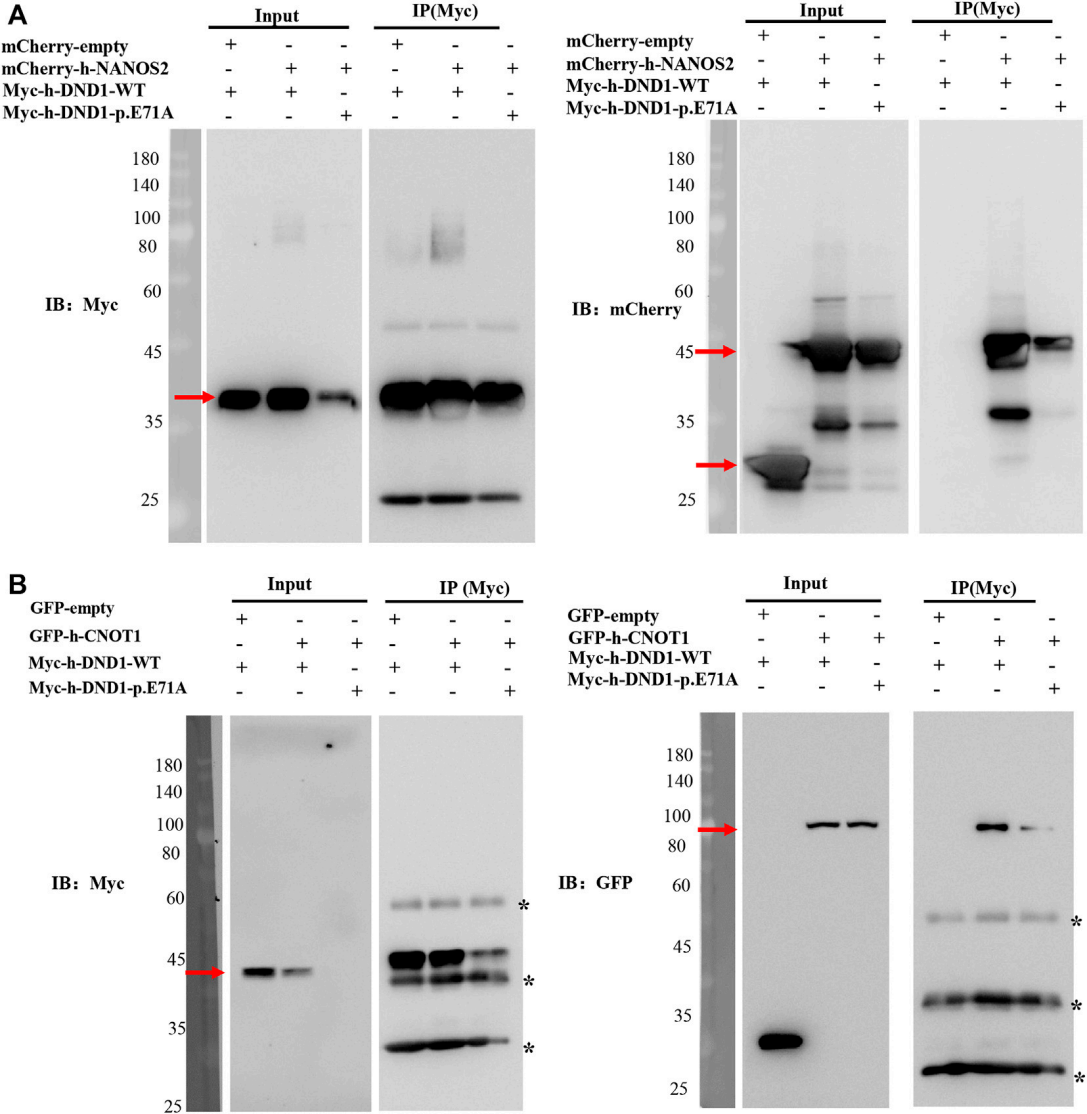
DND1 E71A Mutation disrupted its interactions with NANOS2. **(A)** HEK293T cells expressing the wild-type or mutant Myc-tagged DND1 protein and mCherry-tagged NANOS2 protein were immunoprecipitated with anti-Myc and immunoblotted with Myc and mCherry antibodies. **(B)** HEK293T cells expressing the wild-type or mutant Myc-tagged DND1 protein and GFP-tagged CNOT1(1-551) protein were immunoprecipitated with anti-Myc and immunoblotted with Myc and GFP antibodies.

Previous studies have shown that DND1 cooperatively functions with NANOS2 in male germ cell development. To test the effects of the missense *DND1* variant on the interaction between NANOS2 and DND1, we conducted a co-IP assay in HEK293T cells transfected with Myc-tagged WT DND1, MT DND1 and mCherry-tagged NANOS2. In line with previous studies, Myc-tagged WT DND1 co-precipitated with mCherry-tagged NANOS2. However, the mutated DND1 (E71A) drastically reduced the interaction with NANOS2 (Figure 4A). We further examined whether mutation of DND1 interacts with CNOT1(1-551-aa), a component of the CCR4-NOT (CNOT) deadenylase complex that directly interacts with NANOS2. Similar to other mutants (E59K, V60M, P76L, G82R), DND1 with the E71A mutation almost did not disrupt the interaction with CNOT1 (Figure 4B) (Li et al., 2018). These findings indicated that the E71A mutation decreases protein stability and protein-protein interactions.

## Discussion

The pathology of NOA is characteristic of testicular failure and aberrant spermatogenesis, including Sertoli cell-only syndrome (SCOS) and maturation arrest. A good determination of germ cell fate during the early development of testes is essential to maintain normal spermatogenesis. In the present study, we first reported that missense mutation in *DND1*, an RNA-binding protein that plays an essential role in primordial germ cells (PGCs) survival, caused NOA in patients with infertility from a Pakistani family. *In silico* analysis showed that the high conservation of the mutated amino acid caused structural changes in the DND1 protein, resulting in disruption of its interaction with other protein partners. Our study confirmed the indispensable role of DND1 in early germ cell development and male fertility.

*DND1* deficiency results in male infertility and testis tumors due to the disruption of normal spermatogenesis. Loss of germ cells was observed in zebrafish, frogs and rodents (Weidinger et al., 2003; Youngren et al., 2005; Horvay et al., 2006). *Dnd1* knockout mice displayed early loss of PGCs *via* the BAX-mediated apoptosis pathway (Cook et al., 2009). Likewise, conditional deletion of *Dnd1* causes testis weight decrease and male sterility by loss of both undifferentiated and differentiated spermatogonia (Niimi et al., 2019). A recent study highlighted that PGCs were completely depleted in mouse embryos carrying *Dnd1* missense mutations located in the RBD1 domain (E59K, V60M, P76L, and G82R) (Li et al., 2018). In the current study, we identified a novel *DND1* variant (E71A) causing azoospermia, similar to previous observations that P76L mutation resulted in the absence of germ cells in the small testes in mice. Based on studies from several vertebrates, we speculate that there is an association between *DND1* missense mutation and NOA in humans, although the characteristics of germ cells in seminiferous tubules were not observed due to a lack of testicular tissues.

*Dnd1* was initially identified as a germ plasm component that functions in PGC migration and survival in zebrafish (Weidinger et al., 2003). Further study confirmed that DND1 was specifically expressed among PGCs to pre-meiotic germ cells in mice (Yamaji et al., 2017). *DND1* transcripts were also enriched in spermatogonia and spermatids in humans (Karlsson et al., 2021), which may suggest a similar role for DND1 in germ cells from humans and mice. More importantly, the localization of DND1 was found in nucleus and germ cell granules or P-bodies in both zebrafish and mice, indicating that DND1 is involved in controlling RNA export and stability. Loss of C-terminal RBD regions alter the subcellular localization of DND1, which suggests that RBDs play an essential role in nucleocytoplasmic shuttling (Kedde et al., 2007). In addition, Li *et al.* reported that four critical loss-of-function mutations located in RBD1 caused DND1 protein misfolding and instability (Li et al., 2018). Similar to previous studies, our study showed that the new DND1 mutation in E71A disrupted the structure of RBD1 and affected either protein expression or subcellular localization, expanding RBD-dependent subcellular localization of DND1 in germ cells.

DND1 is an RBP protein that regulates gene expression by recruiting CCR4-NOT and NANOS2 proteins to target mRNAs in mice (Suzuki et al., 2016; Yamaji et al., 2017; Raisch et al., 2019). Mutating conserved amino acids in RBDs disrupted DND1 protein-protein interactions *in vitro* and *in vivo* (Slanchev et al., 2009; Li et al., 2018; Ruthig et al., 2019). Analyses of the three RBD structures and functions showed that RBD1 acts as the main binding platform required for direct interaction with NANOS2, while RBD2 cooperates with RBD1 (Hirano et al., 2022). In this study, we found that DND1 with the E71A mutation decreased the interaction with the NANOS2 protein, confirming that the RBD1 mutation could affect the interactions.

Overall, this study is the first to identify a missense mutation in *DND1* that disrupts protein folding, decreases protein stability and subcellular localization and affects the protein-protein interactions, leading to NOA in humans. Our findings indicate the essential role of DND1 in maintaining germ cell development and the importance of genetic screening for patients with NOA.

## Data Availability

The data presented in the study are deposited in the National Genomics Data Center (NGDC) repository, accession number PRJCA011527.
