# Supplementary material for: A homozygous missense variant in DND1 causes non-obstructive azoospermia in humans
Source: Front Genet. 2022 Sep 30;13:1017302. doi: 10.3389/fgene.2022.1017302 (PMC9561125; doi:10.3389/fgene.2022.1017302)
Supplement: Supplementary file 1 [file Table1.DOCX]

**Supplementary file 1. Whole exome sequencing (WES) and data analysis**.


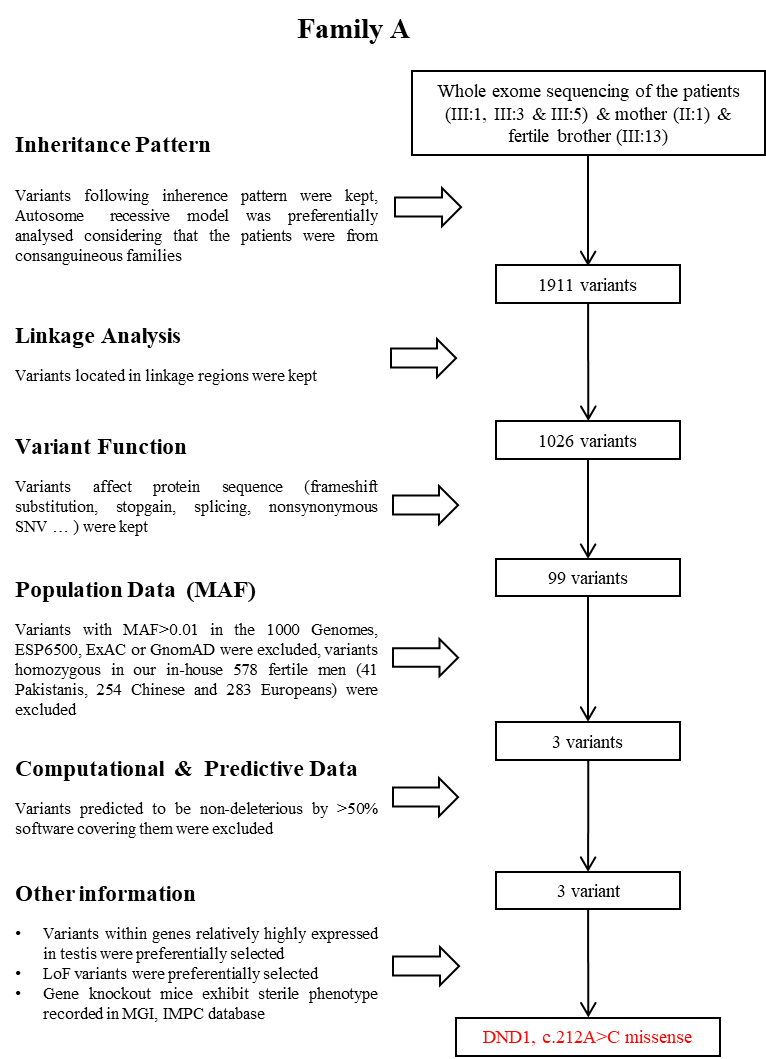


Minor allele frequency (MAF) less than 0.01 was considered for filtration of the existing mutations in the 1000 Genomes, ESP6500, ExAC, and GenomAD databases. (LOD, logarithm of the odds; MAF, minor allele frequency)
