# Supplementary material for: A homozygous missense variant in DND1 causes non-obstructive azoospermia in humans
Source: Front Genet. 2022 Sep 30;13:1017302. doi: 10.3389/fgene.2022.1017302 (PMC9561125; doi:10.3389/fgene.2022.1017302)
Supplement: Supplementary file 2 [file Presentation1.PPTX]

## Slide 1
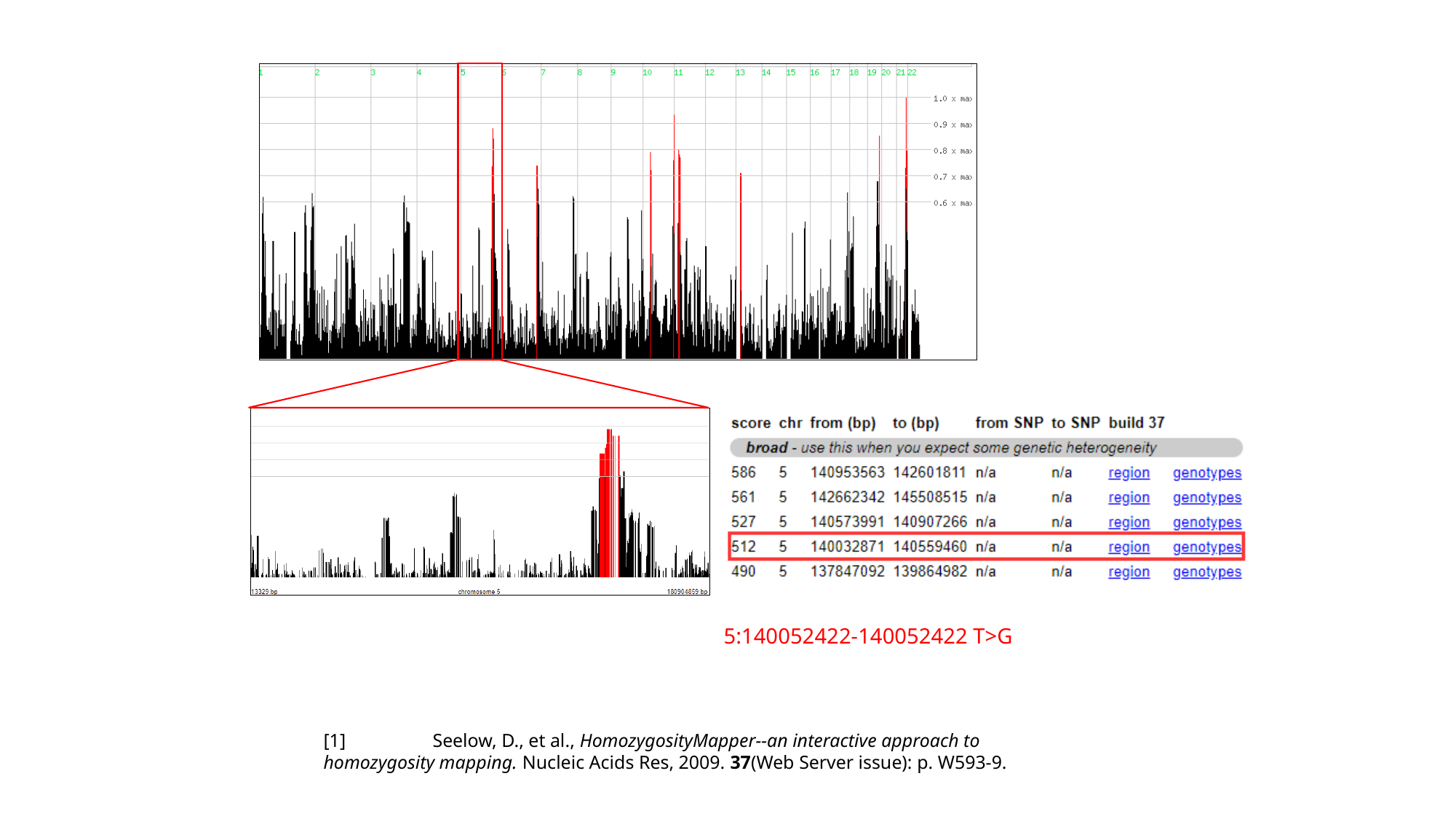

5:140052422-140052422 T>G
[1]	Seelow, D., et al., HomozygosityMapper--an interactive approach to homozygosity mapping. Nucleic Acids Res, 2009. 37(Web Server issue): p. W593-9.
