# Supplementary material for: A homozygous missense variant in DND1 causes non-obstructive azoospermia in humans
Source: Front Genet. 2022 Sep 30;13:1017302. doi: 10.3389/fgene.2022.1017302 (PMC9561125; doi:10.3389/fgene.2022.1017302)
Supplement: Supplementary file 3 [file Table2.DOCX]

**Supplementary file 2: Table of excluded variants and exclusion basis****.**

| **Gene** | **Variants** | **Exclusion basis** |
| --- | --- | --- |
| *PCDHGA8* | NM_032088  c.862C>T  p.Q288X | This gene has a high expression level in thyroid gland, and very low background expression level in spermatogenesis, according to *Fertilityonline* database. |
| *ARHGAP26* | NM_015071  c.2137G>A  p.V713I | According to MGI database, there is no reproductive phenotype reported in Knock out mice of this gene (http://www.informatics.jax.org/allele/MGI:6199115) |
