# Supplementary material for: A homozygous missense variant in DND1 causes non-obstructive azoospermia in humans
Source: Front Genet. 2022 Sep 30;13:1017302. doi: 10.3389/fgene.2022.1017302 (PMC9561125; doi:10.3389/fgene.2022.1017302)
Supplement: Supplementary file 4 [file Table3.DOCX]

**Supplementary file 3**. **Table for Primers used**.

| **Protein** | **Primer** | **Sequences** |
| --- | --- | --- |
| DND1 | GFP-hDND1-F | GAACCGTCAGATCCGCTAGCATGCAGTCCAAGCGGGATTG |
|  | GFP-hDND1-R | TCCTCGCCCTTGCTCACCATCTGTTTAACCATGGTACCTG |
|  | hDND1-mut-F | AGGACGTGTACGCGCACCAGC |
|  | hDND1-mut-R | GCTGGTGCGCGTACACGTCCT |
|  | myc-hDND1-F | CAGAAGCTGATCTCAGAGGAGGACCTGCAGTCCAAGCGGGATTGTGA |
|  | myc-hDND1-R | GATCTAGAGTCGCGGCCGCTTCACTGTTTAACCATGGTAC |
| CNOT1 | GFP-hCNOT1-F | GAACCGTCAGATCCGCTAGCATGAATCTTGACTCGCTCTC |
|  | GFP-hCNOT1-R | TCCTGCAGCTCCACCGCTCGACTGCTCCCCTCTCATGTACC |
| NANOS2 | mCherry-hNANOS2-F | GCATGGACGAGCTGTACAAGATGCAGCTGCCACCCTTCGA |
|  | mCherry-hNANOS2-R | GATCTAGAGTCGCGGCCGCTGCGCTTGACCCTGCGTCCGG |
